# Lower perceived stress among physically active elite private university students with higher levels of gratitude

**DOI:** 10.3389/fspor.2024.1369205

**Published:** 2024-07-09

**Authors:** Laura S. Kabiri, Jennie Le, Cassandra S. Diep, Eunbi Chung, Jacob Wong, Amanda M. Perkins-Ball, Heidi Y. Perkins, Augusto X. Rodriguez

**Affiliations:** Department of Kinesiology, Wiess School of Natural Sciences, Rice University, Houston, TX, United States

**Keywords:** mental health, education, IPAQ, GQ-64, PSS

## Abstract

Elite private universities have high stress levels, particularly for underrepresented populations. While physical activity and gratitude can both reduce stress, independent effects from gratitude and interaction effects between physical activity and gratitude on stress are insufficiently explored. Our study investigated these effects among undergraduates at elite private universities. Undergraduates (*n* = 145) completed an online survey in Fall 2022. Moderate-high physical activity levels were reported by 96.19% of the sample. No significant interaction effect between physical activity and gratitude was seen nor a significant main effect of physical activity on perceived stress. A significant main effect of gratitude on perceived stress [*F* (2, 99) = 16.732, *p* < .001, *ω*2 = .253] was found with higher perceived stress among participants with low compared to moderate (*p* = .001) or high gratitude (*p* < .001). Gratitude exerted an independent, significant effect on perceived stress among elite university undergraduates and could be used as an additional healthy coping mechanism along with physical activity to combat stress.

## 1 Introduction

Stress is particularly common among the undergraduate population. The prevalence of stress and other mental health issues has increased since the COVID-19 pandemic (1). Reasons for this include financial difficulties, academic pressure, and high workloads, as well as the pressure to ideally reflect their cultural communities and the future of mankind (1). While stress is a concern in the general undergraduate population, the issue is particularly worrisome among students in elite private universities, making this population of particular interest (2). Students at these elite academic institutions cite unique issues associated with cultures of perfectionism, academic pressure, and stigmatization of mental illness at higher levels than public institutions of higher education, particularly among first generation, low-income, and underrepresented minority populations on campus (2, 3). Combined with a lack of parental influence and oversight, the increased levels of stress during undergraduate education often lead to unhealthy coping practices.

Binge drinking, as well as tobacco and marijuana use, are all examples of unhealthy coping skills linked to mental health issues among the general undergraduate population (4). Students at private universities also report using alcohol and other substances to cope with academic stress, especially among those who identify as white and of a high socioeconomic background (3). Thus, it is vital to identify and cultivate healthy coping mechanisms for addressing stress during the undergraduate experience, particularly in elite private universities. If developed, these healthy coping skills can extend beyond the university into adulthood (5). However, failure to develop healthy coping skills can transition into work-related stress as adults, with an estimated cost of over $187 billion US dollars annually as lost productivity, health care, and medical costs (6).

Physical activity is one example of a healthy coping skill that improves both physical and mental health. Increased physical activity levels are associated with improvements in cardiorespiratory fitness and muscle mass, as well as decreased risk of cardiovascular disease and related comorbidities like diabetes mellitus and obesity (7). Physical activity helps improve key components of mental health including mood, distress, and mindfulness (8, 9). In addition to improvements in anxiety and depression, physical activity also decreases and even predicts stress (9–11). Regular physical activity helps lower chronic stress hormone levels like cortisol and increases the release of endogenous opiates (12). Combined, these chemical changes help reduce both long-term and short-term stress. Thus, physical activity is an important approach to managing stress and other mental health issues.

Unfortunately, most college students are not getting the recommended amount of physical activity (11). Therefore, its ability to combat stress among this population is limited. The trend in decreased physical activity has been exacerbated since the onset of the COVID-19 pandemic with noted decreases in all levels of physical activity worldwide (13, 14). Reasons for this may include changes in residence and distance to recreational facilities or gyms, as well as a lack of time, including greater time demands from academics and/or supplemental work (13, 14). In addition to a decrease in physical activity, college students are also experiencing increased sedentary time with an average of over 12 h per day spent in sedentary screen time (15). Time spent in inactivity is particularly elevated in overweight and obese students, as well as those from minority groups or raised by parents with lower education levels (15). Thus, while physical activity is known to decrease stress and offers a healthy lifelong coping skill, it is not frequently employed by the undergraduate population.

Like physical activity, gratitude also has positive associations with physical and mental health. For our purposes, gratitude is defined as the belief that we have benefited from good things in the world from sources outside ourselves. In other words, it is “a specific way of thinking about receiving a benefit and giving credit to others besides yourself for that benefit” (16). Physically, gratitude is related to improved inflammatory markers linked to cardiovascular disease, as well as improved sleep (17, 18). Mentally, gratitude is also linked to decreased levels of stress and improved mental health (19). College students who sought mental health treatment and spent three weeks writing letters of gratitude reported significantly fewer anxiety and depression symptoms and better overall function than those who did not (20). Gratitude carries great potential as a coping mechanism for people at high risk for stress-related issues.

Unfortunately, research has yet to determine any independent effect of gratitude or interaction effects from physical activity and gratitude on perceived stress. Therefore, the purpose of this study was to investigate the potential independent and interaction effects of physical activity and gratitude on perceived stress among undergraduates at elite private universities. We hypothesize that physical activity and gratitude will exert independent, inverse effects on stress in private university students with no interaction effect, such that more active, grateful participants show the lowest levels of perceived stress.

## 2 Materials and methods

### 2.1 Participants

The study was approved by the Institutional Review Board at Rice University (IRB-FY2020-327) prior to any recruitment, enrollment, or data collection. The study was part of the larger COVID-19 Stress, Physical Activity, and Nutrition Effects on Students (C-SPANES) II study completed in the fall of 2022. We recruited adults aged 18–25-years-old and currently enrolled in a college or university in the greater Houston area by university email, social media, and chain referral sampling, or encouraging participating students to invite eligible peers to participate. Multiple posts were made over the course of the semester prior to the start of final examinations. Of the over 40 colleges and universities in this area, one was classified as elite (admission rate below 10%) with an undergraduate population of around 4,000 students. This institution hosts both national and international students and awards undergraduate degrees in five major areas of study: natural sciences, engineering, humanities, social sciences, and fine arts. Participants provided informed written consent prior to online survey access. Participants could skip questions or end the survey at any time. For their participation, participants could enter a drawing for a $20 gift card, but no other compensation was provided.

The present study used a subset of data from the larger C-SPANES-II study, including information on physical activity patterns, gratitude, and stress. To address the research question, we excluded participants who were not a current undergraduate at an elite private 4-year university in the Greater Houston area or did not complete the demographics, physical activity, gratitude, and stress portions of the survey.

### 2.2 Protocol

Participants completed an online survey in English, which included basic demographic information like self-reported age, gender, race/ethnicity, and socioeconomic status (SES) as “poor,” “working class,” “middle class,” or “affluent” following best practices (21, 22). We assessed physical activity using seven-day recall through the International Physical Activity Questionnaire—Short Form (IPAQ-SF) (23). The IPAQ-SF is a standard tool that measures physical activity with strong convergent validity (ES*ρ* = .53) (23, 24). We used seven questions from the IPAQ-SF to assess total physical activity. The collected physical activity data was converted to weekly MET-minutes and physical activity categories (low, medium, high) per IPAQ-SF standardized formulas and protocol (23).

We measured gratitude using the Gratitude Questionnaire—Six Item Short Form (GQ-6) (25). The GQ-6 is a self-report tool to assess an individual's gratitude experience in daily life. It has good internal reliability, as well as strong internal consistency with reported Cronbach's alpha values ranging from .78 to 0.82 (25, 26). The GQ-6 also has acceptable discriminant validity with the ability to distinguish gratitude from other positive constructs (e.g., satisfaction with life, vitality, subjective happiness, optimism, and hope) (25). Its brevity and strong psychometric properties make it a standard and popular method of assessing gratitude in a variety of populations, although there may be a ceiling effect affecting the ability to discriminate among individuals who score high on gratitude (27). To address this ceiling effect, gratitude scores were analyzed as scaled classifications of low (6–35 points), moderate (36–40 points), or high (41–42 points) levels of gratitude using existing cut-off scores (25).

Lastly, we used the 10-item Perceived Stress Scale (PSS) to assess perceived stress (28, 29). The PSS is widely used to determine perceived stress with good internal (Cronbach's *α* > .70) and test-retest (>.70) reliability (29–31). A five-point scale (0–4 points) is used for each item, with 4 items reverse scored. The sum of all items creates an overall score with higher values indicating higher perceived levels of stress. Its brevity and ability to distinguish stress from related constructs like anxiety and depression strengthened support for use in this study, particularly among this population (29). Anxiety and depression are also common issues among undergraduates at elite private universities, so it was important to select a tool capable of isolating our desired variable from related covariates (2).

### 2.3 Statistical analysis

We calculated descriptive statistics for all variables and reported them as frequency and mean or percentage ± standard deviation. We cleaned data from the IPAQ-SF before analysis as per the published protocol and recoded cases with physical activity durations <10 min to zero minutes for the corresponding physical activity intensity (32). Gratitude scores and perceived stress scores were also calculated using published protocols (25, 29).

We ran a two-way independent factorial ANOVA to explore interaction and main effects between ordinal physical activity classifications (low, moderate, high) and gratitude levels (low, moderate, high) on perceived stress. Bonferroni *post hoc* tests then explored differences in mean PSS scores between groups. We conducted all statistical analyses using SPSS (version 29, Chicago, IL, USA), with an alpha value of ≤.05 to indicate significance.

## 3 Results

A total of 145 students responded to the C-SPANES–II survey. Of those, 14 did not complete the demographic portion of the survey. Of the remaining 131, seven were not current students at an elite private 4-year undergraduate institution, 12 did not complete the GQ-6, 5 did not have total physical activity scores, and 2 did not complete the PSS. These participants were therefore removed from data analysis based on study inclusion and exclusion criteria, resulting in a final sample of 105 and an overall survey completion rate of 72.41%.

Table 1 includes descriptive statistics for the population. Participants primarily identified as female, Asian, and middle class. The majority were categorized as high physical activity, which reflected the median classification (high physical activity) as well. The average gratitude score was 35.41. Due to the low number of participants classified as low physical activity (*n* = 4), this classification was combined with moderate physical activity to dichotomize the variable for statistical analysis purposes.

**Table 1:** Descriptive characteristics of the entire sample.

Characteristic or demographic	Frequency (*n*)	Mean ± SD or percent
Age (years)	105	19.85 ± 1.36
Gender		
Man/male/masculine	43	40.95
Woman/female/feminine	58	55.23
Gender nonconforming, other	3	2.85
Prefer not to answer	1	0.97
Race		
Asian	46	43.81
Black or African American	12	11.43
Hispanic, Latino, or Spanish Origin	7	6.67
Middle Eastern or North African	2	1.90
White	25	23.81
Persons of multiple races	13	12.38
Socioeconomic status (SES)		
Poor	3	2.86
Working class	14	13.33
Middle class	67	63.81
Affluent	21	20.00
Physical activity (PA)		
Average PA (total MET-min/week)	105	3,576.30 ± 2,186.45
Low physical activity classification	4	3.81
Medium physical activity classification	45	42.86
High physical activity classification	56	53.33
Gratitude		
Average GQ-6 score	105	35.41 ± 5.73
Low gratitude classification	43	40.95
Moderate gratitude classification	38	36.19
High gratitude classification	24	22.86

**Figure 1:**
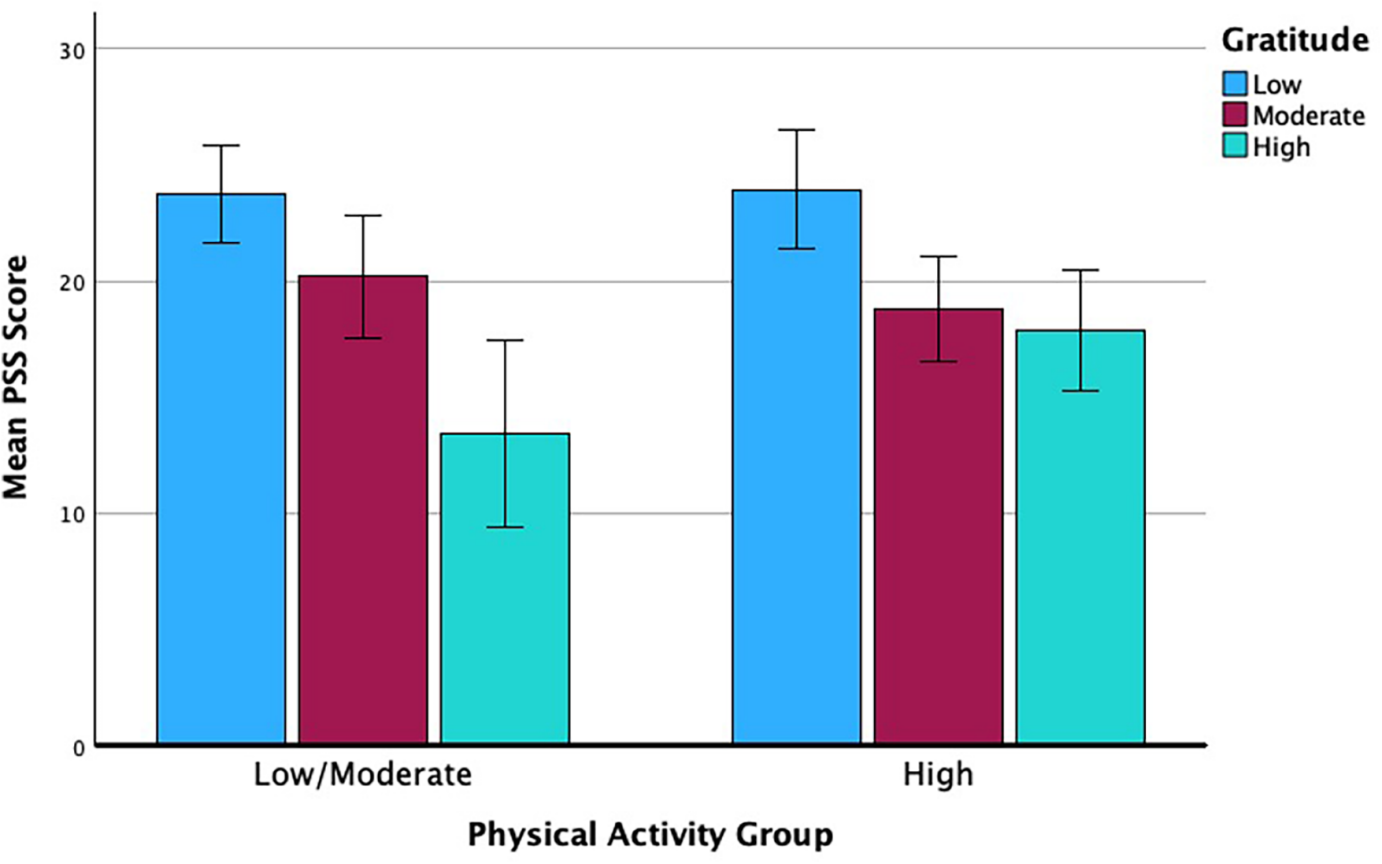
Means PSS scores by PA and GQ-6 classifications (error bars: 95% CI).

No clear violations of the linear model based on histograms and scatterplots of the residuals were seen. No significant main effect of physical activity [*F* (1, 99) = .930, *p* = .337, *ω*2 = .009] was found, nor a significant interaction between physical activity and gratitude [*F* (2, 99) = 1.930, *p* = .151, *ω*2 = .038], on perceived stress. However, there was a significant main effect of gratitude on perceived stress with a large effect size [*F* (2, 99) = 16.732, *p* < .001, *ω*2 = .253]. Bonferroni *post hoc* tests revealed significantly higher levels of perceived stress among students with low levels of gratitude compared to those with moderate (*p* = .001) or high levels of gratitude (*p* < .001), but no significant difference in perceived stress between students with moderate and high levels of gratitude (*p* = .140). Figure 1 shows the means by grouping.

In addition to no significant main effect between physical activity and perceived stress, there was also no significant difference in gratitude scores between the two physical activity groups. Gratitude scores were similar in both low/moderately active and highly active students. Overall, students classified as highly active with low levels of gratitude showed the highest mean perceived stress, while low/moderately active students with high levels of gratitude reported the lowest perceived stress. Additional details and PSS scores by subgroup can be seen in Table 2.

**Table 2:** Means and standard deviations for PSS scores by PA and GQ-6 classifications.

	Low/moderate physical activity classification			High physical activity classification		
	*M*	SD	*n*	*M*	SD	*n*
Low gratitude group	23.72	4.968	26	23.94	6.129	17
Moderate gratitude group	20.19	4.929	16	18.82	5.315	22
High gratitude group	13.43	3.409	7	17.88	6.092	17
Overall gratitude	21.10	5.875	49	20.09	6.265	56

## 4 Discussion

Our findings show no interaction effect such that physical activity and gratitude exert independent effects on perceived stress in private university students. However, only gratitude exerted a significant effect on perceived stress. Our original hypothesis that both physical activity and gratitude would significantly and independently impact perceived stress was partially supported. The non-significant interaction effect suggests that physical activity and gratitude impact perceived stress in unrelated ways. Thus, they can be considered separate methods of addressing perceived stress with distinct, unique effects. Our findings support existing literature which also shows no relationship between the two constructs (33, 34). However, physical activity and gratitude can and do overlap. A recent science review concluded that gratitude positively impacts adherence to health behaviors like physical activity (17). Moreover, gratitude also has pro-social benefits promoting relationships and human connection (35). These pro-social benefits may also encourage people to seek out group exercise classes and other activity programs with social components. While there is no significant interaction effect in our study, both physical activity and gratitude offer complementary benefits to addressing perceived stress.

First, we saw no significant impact of physical activity on perceived stress among our sample. However, this does not negate the benefits of physical activity on stress which have been well established in the literature (8, 9). In fact, a previous study by our same research group concluded that not only were physical activity and stress related, but physical activity was also a significant predictor of perceived stress in a similar undergraduate population in elite academic institutions (10). The chemical changes seen with regular physical activity are also well established in the literature (12). It is widely accepted that physical activity can decrease general cortisol levels, which has multiple health benefits including lower overall stress and improved sleep (36). Of note, 96.19% of participants in our sample were classified as moderately or highly active. Therefore, it is possible that because most of our sample was already engaging in regular physical activity, they were already receiving the stress-reducing benefits known to accompany exercise which would explain the lack of a significant independent effect of physical activity on stress.

Second, gratitude classification independently and inversely impacted perceived stress in our study. Students with higher levels of gratitude also perceived less stress regardless of physical activity level which suggests that gratitude as a coping mechanism may decrease perceived stress regardless of exercise. In the literature, we also can find support for a relationship between gratitude and lower stress (16, 37). Likewise, gratitude has been associated with endogenous mu-opioid peptides and receptor systems, which are proffered as potential explanations for the pro-social, positive affect, and stress reduction seen in high levels of gratitude (35). Neurologically, gratitude has very specific links to stress. Gratitude involves the medial prefrontal cortex of the brain where the two hemispheres align and is involved in prosocial behavior which helps build meaningful connections and relationships among humans and primates (16, 19). It also regulates emotions and supports the stress relief process, which may explain why individuals with high levels of gratitude tend to experience lower stress and greater life satisfaction (19, 37). In one study, practicing gratitude led to structural changes in the frontal lobe itself (19). It also helps the body and brain both to relax and “destress” (19). In our study, the inverse and independent impact of gratitude on perceived stress is noteworthy. Since our population was largely physically active, gratitude offers a new area of focus as an additional healthy coping mechanism for people in high-stress environments. In other words, gratitude may be an effective complement for stress management in people who are already receiving the mental and physical health benefits of regular exercise, yet still struggle with high levels of perceived stress.

Altogether, our findings suggest the importance of gratitude practices in managing stress among elite private university students, particularly those who are already physically active. Notably, simple interventions exist to improve both physical activity and gratitude through purposeful practice. Options for increasing physical activity are practically limitless and range from competitive athletics to co-ed sports to simple walking programs. Many are low to no-cost and require minimal upfront investment. These activities are widely available and can be adopted as healthy coping mechanisms to combat stress throughout adulthood. Likewise, journaling is one of the most effective means of increasing gratitude (16). Simple exercises in gratitude journaling implemented in schools show multiple positive impacts. In one study, early adolescents who journaled daily, listing five items for which they were grateful, reported more optimism and fewer negative emotions and physical complaints. Moreover, this benefit lasted at least three weeks past the intervention (38). However, not all people who practice gratitude feel fewer negative emotions like anxiety, depression, and stress, but they do report an increase in positive emotions like optimism, pleasure, enthusiasm, and joy (16). In this way, gratitude offers a healthy way to process and help offset negative emotions (39). Regardless, gratitude is a simple and effective way to help combat feelings of perceived stress.

### 4.1 Study limitations

Limitations of our study include a small and homogenous sample of physically active private university students, so generalizability to the population at large or to more diverse undergraduate and adult populations may be limited. However, our population of predominantly female, white or Asian, and middle-to-upper class SES is reflective of elite private university students in the United States (40–42). Moreover, this research study employed a cross-sectional design with no control group and, as such, cannot determine causality. Future studies should employ purposeful interventions and collect longitudinal data to strengthen and clarify our findings. Finally, data for this study were collected by online survey using self-report tools, introducing the potential for self-selection and self-reporting bias. However, the tools used in this study, as well as the online survey and sampling strategies, are widely accepted and used techniques in social and behavioral research.

### 4.2 Conclusion

In short, our findings showing no interaction effect between physical activity and gratitude, as well as an inverse, independent effect of gratitude on perceived stress, are new and novel. Universities and health professionals could use these results to provide additional healthy coping mechanisms for managing stress. While our results have implications for adults in general, our findings are particularly important in high-stress populations like students enrolled in elite academic institutions and those from first generation, low-income, and underrepresented minority populations. While both physical activity and gratitude can be impactful as solo constructs, it is possible that encouraging physically active adults, who still struggle with stress management, to adopt simple gratitude practices may further help alleviate perceived stress. Future studies should investigate the longitudinal effects of physical activity and gratitude on perceived stress and employ experimental designs to help determine causality and clarify impact.

## Data Availability

The datasets presented in this article are not readily available because data sharing is only considered upon written request. Requests to access the datasets should be directed to laura.kabiri@rice.edu.
